# Comparison of direct cDNA and PCR-cDNA Nanopore sequencing of RNA from Escherichia coli isolates

**DOI:** 10.1099/mgen.0.001296

**Published:** 2024-10-25

**Authors:** Gillian Rodger, Samuel Lipworth, Lucinda Barrett, Sarah Oakley, Derrick W. Crook, David W. Eyre, Nicole Stoesser

**Affiliations:** 1 Nuffield Department of Medicine, University of Oxford, Oxford, UK; 2 NIHR Health Protection Unit in Antimicrobial Resistance and Healthcare-associated Infection, University of Oxford, Oxford, UK; 3 Oxford University Hospitals NHS Foundation Trust, Oxford, UK; 4 NIHR Oxford Biomedical Research Centre, University of Oxford, Oxford, UK; 5 Big Data Institute, Nuffield Department of Public Health, University of Oxford, Oxford, UK

**Keywords:** antimicrobial resistance gene expression, bacterial transcriptomics, *Escherichia coli*, long-read RNA-Seq, nanopore sequencing

## Abstract

Whole-transcriptome (long-read) RNA sequencing (Oxford Nanopore Technologies, ONT) holds promise for reference-agnostic analysis of differential gene expression in pathogenic bacteria, including for antimicrobial resistance genes (ARGs). However, direct cDNA ONT sequencing requires large concentrations of polyadenylated mRNA, and amplification protocols may introduce technical bias. Here we evaluated the impact of direct cDNA- and cDNA PCR-based ONT sequencing on transcriptomic analysis of clinical *Escherichia coli*. Four *E. coli* bloodstream infection-associated isolates (*n*=2 biological replicates per isolate) were sequenced using the ONT Direct cDNA Sequencing SQK-DCS109 and PCR-cDNA Barcoding SQK-PCB111.24 kits. Biological and technical replicates were distributed over eight flow cells using 16 barcodes to minimize batch/barcoding bias. Reads were mapped to a transcript reference and transcript abundance was quantified after *in silico* depletion of low-abundance and rRNA genes. We found there were strong correlations between read counts using both kits and when restricting the analysis to include only ARGs. We highlighted that correlations were weaker for genes with a higher GC content. Read lengths were longer for the direct cDNA kit compared to the PCR-cDNA kit whereas total yield was higher for the PCR-cDNA kit. In this small but methodologically rigorous evaluation of biological and technical replicates of isolates sequenced with the direct cDNA and PCR-cDNA ONT sequencing kits, we demonstrated that PCR-based amplification substantially improves yield with largely unbiased assessment of core gene and ARG expression. However, users of PCR-based kits should be aware of a small risk of technical bias which appears greater for genes with an unusually high (>52%)/low (<44%) GC content.

**Impact Statement**

RNA sequencing (RNA-Seq) allows quantification of RNA within a biological sample providing information on the expression of genes at a particular time. This helps understand the expression of antimicrobial resistance genes (ARGs). In RNA-Seq experimental workflows extra steps of reverse transcription may be needed to generate more stable cDNA to allow for amplification by PCR if starting RNA input was low. Two current methods of long-read RNA-Seq include direct cDNA- and PCR-cDNA-based sequencing (Oxford Nanopore Technologies, ONT). However, few studies have compared these two methods of RNA-Seq using clinical bacterial isolates [7]. We therefore undertook a study to compare both kits using a methodologically balanced design of biological and technical replicates of *Escherichia coli*. Our study showed that direct cDNA and PCR-cDNA sequencing is highly reproducible between biological and technical *E. coli* replicates with very small differences in gene expression signatures generated between kits. The PCR-cDNA kit generates increased sequencing yield but had a smaller proportion of mappable reads, with more shorter reads of lower quality and some PCR-associated bias. PCR-based amplification greatly increased sequencing yield of core genes and ARGs, but there may be a small risk of PCR bias in genes that have a higher GC content.

## Data Summary

The transcript reads of the four sequenced *Escherichia coli* strains have been deposited in the Figshare, DOI: 10.6084/m9.figshare.25044051. Sequencing data have been deposited in NCBI BioProject accession: PRJNA604975; Isolate A SRR13991775, Isolate B SRR13992036, Isolate C SRR13993239, Isolate D SRR13991696. The authors confirm all supporting data (available in Figshare), code (available at: https://github.com/samlipworth/rna_methods) and protocols have been provided within the article or through supplementary data files.

## Introduction

RNA sequencing (RNA-Seq) has become the leading method for transcriptome-wide analysis of differential gene expression (DGE) [1,3]. RNA-Seq can characterize all transcripts over a large dynamic range; quantify terminator efficiency and small RNAs; and measure transcriptional abundance, including operons, whilst being cost-effective [4,6]. Having a greater understanding of bacterial gene expression would provide valuable insight into the relationship between genotype and phenotype for diverse microbial functions, including antimicrobial resistance (AMR).

To date, most RNA-Seq has been based on short-read Illumina sequencing generating read lengths up to 300 bp, and short-read RNA-Seq workflows and computational tools have evolved substantially [2,5]. During library preparation for short-read sequencing, however, RNA is fragmented prior to reverse transcription (RT), potentially resulting in lost information during read mapping and making distinguishing between overlapping transcripts and therefore full-length transcript analysis challenging [7,8]. Recent advances in long-read sequencing, for example from Oxford Nanopore Technologies (ONT; hereafter referred to as ‘nanopore’), have removed the need for RNA fragmentation thus permitting sequencing of longer transcripts, and improved read-mapping strategies and transcript identification [7,9].

Nanopore RNA-Seq protocols reflect two main approaches. The first is direct sequencing of RNA molecules using the RNA-Seq kit [SQK-RNA002, upgraded to SQK-RNA004 in early 2024 for compatibility with the latest Early Access program RNA (FLO-MIN004RA) flow cell] [7,8, 10]. The second incorporates cDNA synthesis allowing enrichment of full-length sequenced transcripts [7]. For cDNA sequencing, nanopore offers two kits – a direct cDNA sequencing kit (SQK-DCS109, evaluated in this study and hereafter referred to as the ‘direct’ kit; replaced by SQK-LSK114 in mid-June 2023) and a kit which includes a cDNA PCR-based amplification step (SQK-PCB111.24, evaluated in this study and hereafter referred to as the ‘PCR’ kit; pending replacement by SQK-PCB114.24 currently an Early Access product, fully available in March 2024). Although direct cDNA sequencing could avoid PCR amplification bias, it also requires a high polyadenylated mRNA input (>100 ng) and the library preparation time is longer (~5 h vs 1 h 45 min).

Bacterial RNA-Seq workflows can be challenging as mRNA typically represents <5% of RNA isolated. Most RNA extracted is rRNA (i.e. 5S, 16S, 23S) which requires depletion to allow for sufficient, cost-effective mRNA sequencing coverage [11,12], but poor sequence quality can result from RNA degradation during depletion. Moreover, bacterial mRNA is not naturally polyadenylated at the 3′ end, which is a prerequisite for nanopore protocols where polyadenylated mRNA is annealed to an oligo(dT) primer for PCR-cDNA sequencing or ligated to a double-stranded oligo(dT) splint adapter in direct RNA sequencing. Given large concentrations of polyadenylated mRNA are required for nanopore sequencing, optimization of extraction and template preparation workflows would be ideal [7,13]. Additionally, computational tools are generally designed for eukaryotic transcriptomics or for short-read prokaryotic transcriptomics, and a bioinformatics pipeline for analysing long-read bacterial transcriptome data would be beneficial [14,15]. Furthermore, it is essential to capture the impact of biological and experimental variability on nanopore RNA-Seq outputs, using both biological and technical replicates [16], as batch effects could result in the misidentification of differentially expressed genes [3,14, 15, 17].

Here we compared the nanopore direct and PCR sequencing kits. Given the global importance of AMR in *Escherichia coli* [18] and the potential for ONT RNA-Seq to improve our ability to predict phenotype from genotype, we chose to include clinical isolates carrying multiple AMR-associated genes and have included a focus on the impact of these kits on analysis of their expression. We evaluated four clinical isolates of *E. coli*, assessing biological and technical batch effects and evidence of PCR bias with PCR-amplified cDNA libraries. Our work builds on previous experimentation on a single laboratory strain of *E. coli* [7].

## Methods

### Isolates for testing and bacterial growth curves

We investigated four *E. coli* bloodstream infection-associated isolates stored as stocks at −80 °C in 10% glycerol nutrient broth; these isolates had all demonstrated amoxicillin-clavulanate (co-amoxiclav) and ceftriaxone resistance using the BD Phoenix and EUCAST clinical breakpoints [19].

### RNA extraction

Prior to implementing the final optimized extraction and sequencing workflow described below, we analysed the outputs of several RNA extraction kits, mRNA enrichment/rRNA depletion kits, polyadenylation approaches and ONT sequencing kits, including: PureLink RNA Mini kit (Thermo Fisher Scientific), MICROBExpress Bacterial mRNA Enrichment Kit, Poly(A) Polymerase Tailing Kit (Lucigen), Direct RNA Sequencing Kit SQK-RNA002, and Direct cDNA Sequencing Kit SQK-DCS109 single-plex and multiplexed using EXP-NBD104. These approaches were not used in the final workflow because of a combination of poor RNA yield post-extraction, lengthy incubation times during mRNA enrichment and polyadenylation, inability to multiplex sequencing reactions (RNA002 kit) and poor overall sequencing outputs with these combinations. Details of the methodology and sequencing outputs are included in Table S1 (available in the online version of this article); we have included this information so that other research teams are aware of strategies we evaluated that worked less well.

Single colonies of each of the *E. coli* isolates were inoculated into 10 ml of Luria-Bertani medium (LB; i.e. without antibiotics) and grown overnight at 37 °C with shaking at 160 r.p.m. A 1 : 100 dilution of the overnight inoculum was sub-cultured in 10 ml LB and grown to mid-log phase (0.5 at OD_600nm_) for RNA extraction. RNA was extracted from biological replicates (*n*=2 for each * E. coli* isolate) on the automated KingFisher Flex platform using the MagMax Viral/Pathogen II Nucleic Acid Isolation Kit (Thermo Fisher Scientific). Post-extraction DNase treatment was performed using the TURBO DNA-*free* Kit (Invitrogen) according to the manufacturer’s instructions. Total RNA quality and integrity were assessed using the TapeStation 4200 (Agilent Technologies) (Table S2) and quantified using the Broad Range RNA Qubit kit (Thermo Fisher Scientific). RNA with a RIN (RNA integrity number) >7 was used [20]. rRNA depletion was performed using the QIAseq FastSelect 5S/16S/23S kit (Qiagen) in accordance with the manufacturer’s instructions with the addition of RNase inhibitor (New England BioLabs) incorporated into each reaction. The addition of poly(A) tails to mRNA transcripts was performed using the Nanopore recommended protocol [21] using Poly(A) Polymerase (New England BioLabs) with a 1 min incubation time and included the addition of RNase inhibitor (New England BioLabs) in each reaction. A final clean-up of mRNA using RNAClean XP beads (Beckman Coulter) was performed prior to library preparation. mRNA was checked pre- and post-poly(A) tail addition using an Agilent RNA 6000 Nano kit run on a 2100 BioAnalyser (Agilent Technologies) (Fig. S1). Quantifications were performed using the High Sensitivity RNA Qubit kit (Thermo Fisher Scientific). To alleviate any possible degradation of RNA from repeated free/thaw cycles, the steps from extraction to cDNA synthesis were completed in a single day.

### RNA library preparation and sequencing

**Fig. 1.**
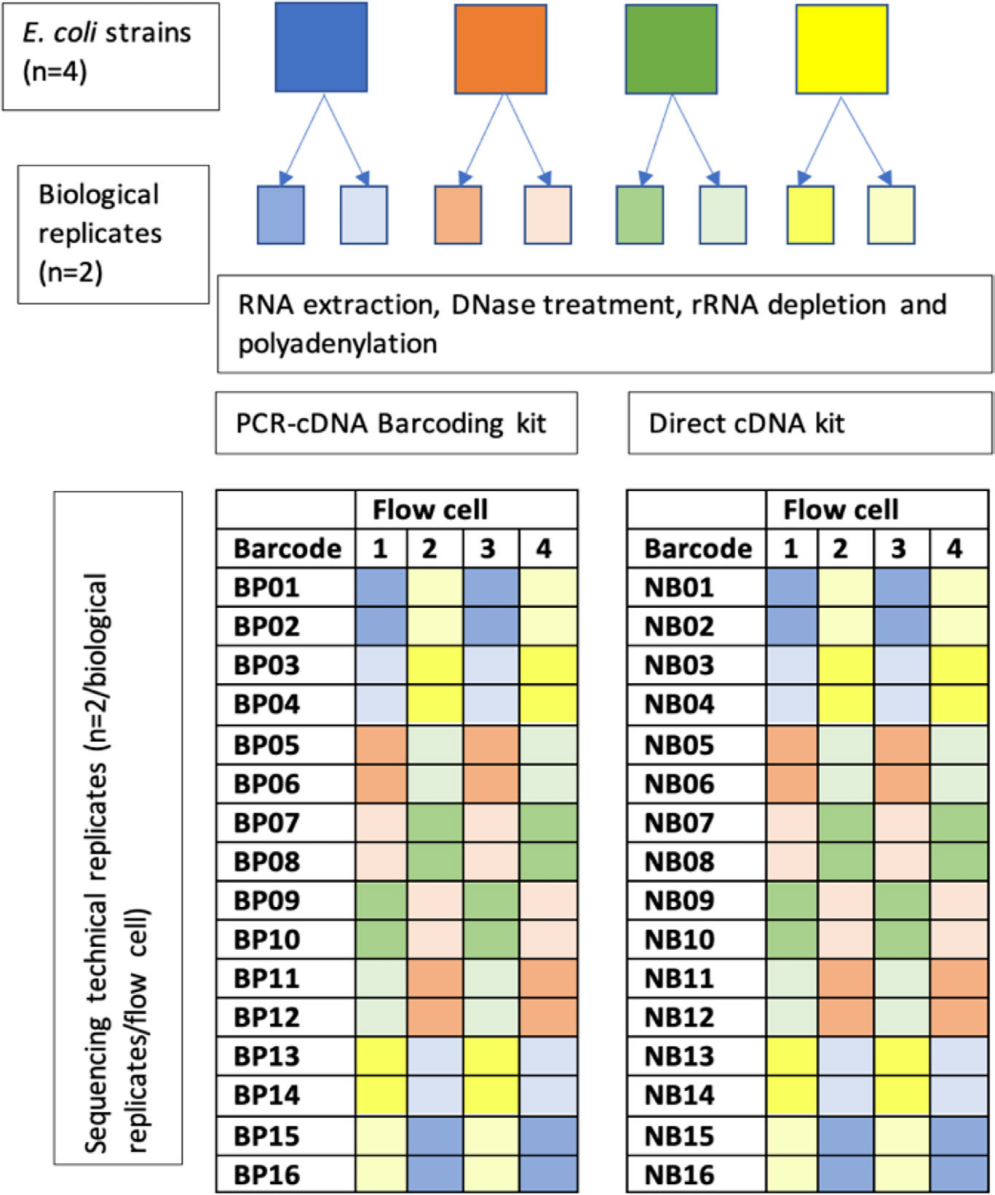
Experimental design illustrating the number of biological and technical replicates sequenced per flow cell and per kit.

Two sequencing kits were selected for comparison in the final workflow: Direct cDNA Sequencing SQK-DCS109 and PCR-cDNA Barcoding SQK-PCB111.24 with libraries prepared in accordance with the manufacturer’s instructions. Our experimental design tested differential expression between *E. coli* (*n*=4 isolates, *n*=2 biological replicates per isolate) using a balanced block design [16]. Technical replicates (*n*=2 per biological replicate) were multiplexed (*n*=16 replicates per flow cell in total) across four flow cells per sequencing kit (i.e. *n*=8 flow cells in total; Fig. 1). Nanopore libraries were sequenced using R9.4.1 (FLO-MIN106) flow cells on a GridION with MinKNOW software v21.11.7 and basecalled using Guppy (v6.1.5) for the maximum 72 h run time.

### DNA extraction and sequencing

Short-read sequencing data (Illumina HiSeq) were created as part of a previous project [22] (NCBI BioProject accession: PRJNA604975; Isolate A SRR13991775, Isolate B SRR13992036, Isolate C SRR13993239, Isolate D SRR13991696; all ENA run accessions). DNA was extracted for nanopore sequencing using the automated KingFisher Flex platform using the MagMax Viral/Pathogen II Nucleic Acid Isolation Kit (Thermo Fisher Scientific). DNA was sequenced on an ONT GridION using the rapid barcoding kit (SQK-RBK004) R9.4.1 (FLO-MIN106) flow cells with MinKNOW software v21.11.7 for 48 h run time.

### Sequence analysis

Raw fast5 files were basecalled and demultiplexed using Guppy (v6.1.5). Only reads that passed the default ONT QScore threshold were included in subsequent analysis (min Q score of reads analysed 8.98). Sequence length and quality statistics were extracted using NanoStat (v1.4.0). Hybrid assemblies were created using Unicycler (--mode bold) and subsequently annotated with Bakta (v1.6.1) to create transcript references. Reads were then mapped to the transcript reference using Minimap2 (-ax splice, otherwise default settings v2.17-r941) [23]. In addition to the efforts to deplete rRNA in the laboratory workflow described above, reads mapping to rRNA coding sequences were discarded from further analysis (Fig. S2). Transcript abundance was quantified using Salmon (v1.8.0) [24]. Acquired AMR genes were identified using AMRFinder (v 3.11.2) [25]. We opted to focus on acquired AMR genes (rather than, for example, chromosomal efflux pumps present in other databases such as MegaRes) because they are more likely to be associated with clinically significant changes in minimum inhibitory concentration (MIC) of first-line antibiotics. NanoPlot [26] was used to assess the relationship between read quality and length after randomly downsampling sequenced data to ~250 Mb using Rasusa [27] for computational feasibility. Multi-locus sequence typing (MLST) was performed *in silico* using the MLST tool [28,29]. Assemblies were assessed using the CheckM tool (v1.2.2, default settings) [30], which correctly predicted that all isolates belonged to marker lineage ‘f_Enterobacteriaceae’ and confirmed that all were probably of adequate quality [completeness 99.97% for all and contamination median 0.39% (range 0.33–0.46%)].

### Statistical analysis

EdgeR (v3.42.2) was used to fit quasi-likelihood negative binomial generalized log-linear models (glmQLFit), utilizing a design matrix incorporating both biological and technical replicates, to these count data (after filtering using filterByExpr min.count=1) to compare, for example, expression between direct and PCR kits. *P*-values were adjusted for multiple comparisons using a false discovery rate adjustment [i.e. p.adjust(method=‘fdr’)] with values <0.05 considered as ‘significant’. Distributions (e.g. of read quality scores) between groups were compared using Kruskal–Wallis tests and correlations using Spearman’s coefficient. Proportions between groups in 2×2 tables were compared using Fisher’s exact tests. All statistical analysis was performed in R v4.2.1 [31].

## Results

### Description of isolates

The four isolates used in this study were identified as belonging to sequence types (STs) 131 (A, C), 1193 (D) and an unclassified ST (B). A total of 36 AMR genes (ARGs) were identified: 12 in isolate A, 7 in isolate B, 7 in isolate C and 10 in isolate D (Table 1). The reference transcript sizes for each isolate were: Isolate A – 5425 coding sequences (4 758 606 bp), Isolate B – 5570 coding sequences (4 830 938 bp), Isolate C – 5523 coding sequences (4 809 970 bp) and Isolate D – 5191 coding sequences (4 564 879 bp).

**Table 1.** Summary of isolate assemblies and ARGs detected

Isolate	Contig	ARGs
A	Chromosome – 5 216 957 bp	2 copies *bla*_CTX-M-15_1 copy *aac(3)-IIe*, *catB3*, *bla*_OXA-1_, *aac(6′)-Ib-cr5*, *tet(A*), *bla*_TEM-1_, *dfrA14*, *sul2*, *aph(3″)-Ib*, *aph(6)-Id*
	Plasmid 1 – 113 482 bp	–
	Plasmid 2 – 4236 bp	–
B	Chromosome – 5 174 320 bp	–
	Plasmid 1 – 138 864 bp	*tet(B*), *sul1*, *aadA5*, *dfrA17*
	Plasmid 2 – 97 597 bp	*tet(B*), *aph(3″)-Ib*, *aph(6)-Id*
	Plasmid 3 – 7940 bp	–
	Plasmid 4 – 5631 bp	–
	Plasmid 5 – 4237 bp	–
	Plasmid 6 – 4072 bp	–
	Plasmid 7 – 2101 bp	–
	Plasmid 8 – 1552 bp	–
C	Chromosome – 5 113 180 bp	–
	Plasmid 1 – 116 873 bp	*dfrA17*, *tet(A*), *aph(6)-Id*, *aph(3″)-Ib, sul2*
	Plasmid 2 – 87 246 bp	–
	Plasmid 3 – 71 559 bp	–
	Plasmid 4 – 8957 bp	*mph(A*), *bla*_CTX-M-27_
	Plasmid 5 – 5631 bp	–
	Plasmid 6 – 5221 bp	–
	Plasmid 7 – 5166 bp	–
	Plasmid 8 – 4082 bp	–
	Plasmid 9 – 1718 bp	–
D	Chromosome – 4 959 734 bp	*bla*_CTX-M-15_, *aac(6′)-Ib-cr5*, *bla*_OXA-1_, *catB3*, *aac(3)-IIe*
	Plasmid 1 – 92 583 bp	*sul2*, *aph(3″)-Ib*, *aph(6)-Id*, *sul1*, *dfrA5*
	Plasmid 2 – 79 495 bp	–

### The PCR kit produces a greater sequencing yield but with shorter read lengths and lower quality scores

The total data yield averaged across four flow cells after 72 h and multiplexing 16 barcoded samples was 1.8 and 11.0 Gb for the direct and PCR kits, respectively. However, median read lengths produced by the direct kit were longer than those produced by the PCR kit [501 bp (interquartile range, IQR: 390–603) vs 318 bp (IQR: 293–400); Kruskall–Wallis *P*<0.001] (Fig. S3). Read quality (Fig. S4) was broadly comparable between kits, though some difference in Q-score distributions was observed (Kolmogorov–Smirnov test D=0.27, *P*<0.001) with slightly higher values obtained for the direct versus PCR kit [median Q-score: 12.0 (IQR: 10.3–13.9) vs 11.2 (IQR: 10.4–12.1), *P*<0.001].

### Mappable reads are longer and of higher quality and represent a greater proportion of total reads in the direct kit

Overall, the percentage of reads that could be mapped to the respective reference transcript was higher for the direct versus the PCR kit [median mapped percentage: 85% (IQR: 77–88%) vs 47% (IQR: 41–55%), *P*<0.001]. Using the direct kit, there was no difference in the percenatge of reads that could be mapped between isolates [Isolate A median: 83% (IQR: 82–88) reads mapped, Isolate B median: 86% (IQR: 80–87) reads mapped, Isolate C median: 83% (IQR 76–89) reads mapped, Isolate D median: 85% (IQR: 82–88) reads mapped; *P*=1.00], but this was not the case for the PCR kit [Isolate A median: 48% (IQR: 42–55) reads mapped, Isolate B median: 46% (IQR: 39–51) reads mapped, Isolate C median: 61% (IQR: 48–64) reads mapped, Isolate D median: 41% (IQR 38–47) reads mapped; *P*=0.02, Fig. S5).

For both the direct and PCR kits, reads that could be mapped were longer [direct mapped median read length: 510 bases (IQR: 388–608) vs direct unmapped median read length: 361 bases (IQR: 298–466), *P*<0.001 and PCR mapped median read length: 437 bases (IQR: 342–569) vs unmapped median read length: 301 bases (IQR: 283–322), *P*<0.001]. They were also of higher quality [direct mapped median Q score: 12.4 (IQR: 10.4–14.3) vs direct unmapped median Q score: 11.2 (IQR: 10.1–12.8), *P*<0.001 and PCR mapped median Q score: 11.8 (IQR 10.9–12.7) vs unmapped median Q score: 11.0 (IQR: 10.2–11.8), *P*<0.001] than those that could not be mapped (Fig. S6).

### Read counts are strongly correlated for biological replicates and kits

**Fig. 2.**
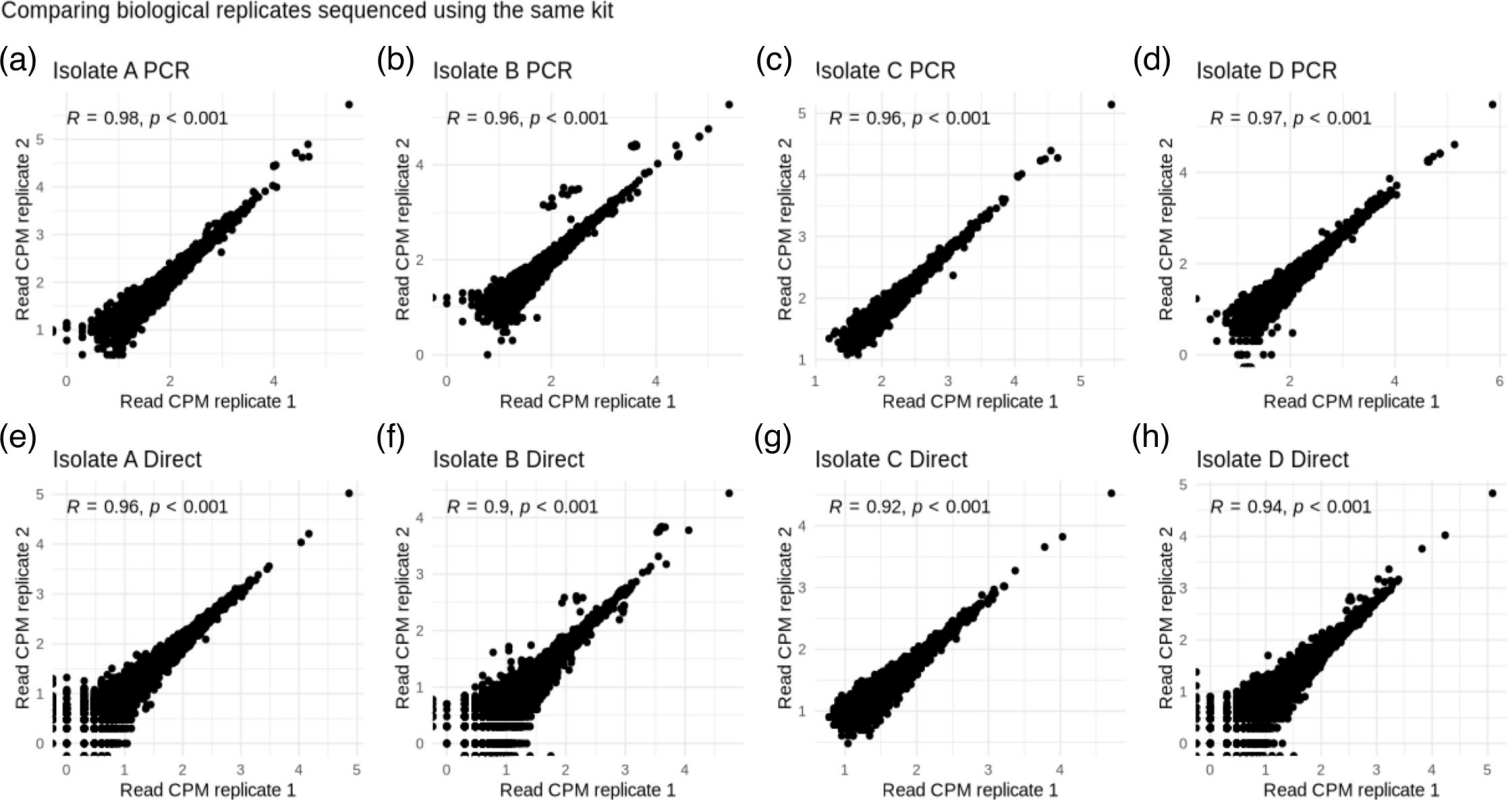
Read count correlations when comparing: (**a–d**) biological replicates sequenced with the PCR kit, and (e–h) biological replicates sequenced using the direct kit. CPM, log_10_ counts per million. Spearman correlation coefficients and *P*-values are shown within each plot. For this analysis read counts were summed across technical replicates.

**Fig. 3.**
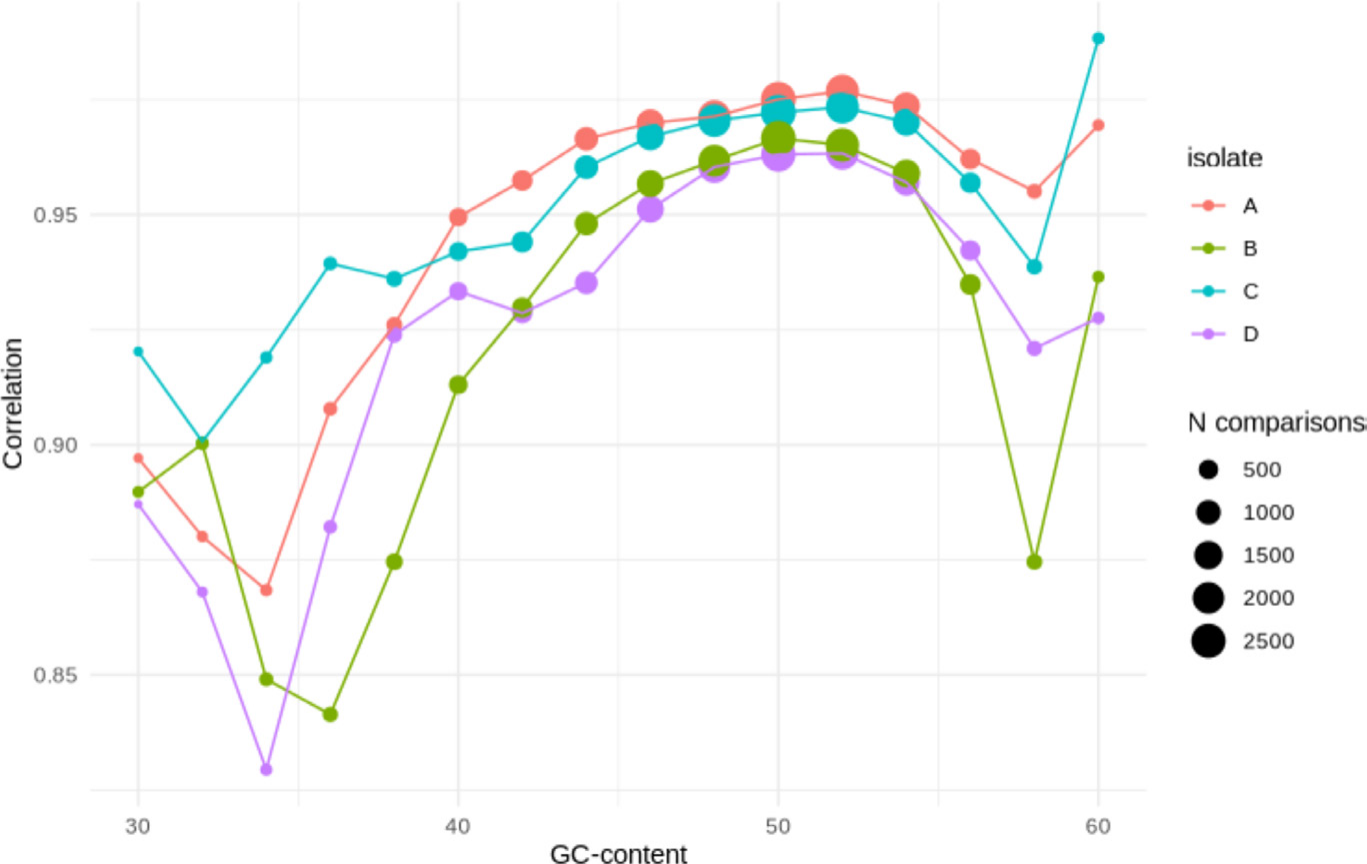
Read count correlation between the PCR and direct kits for genes with different GC content (%).

For *n*=3786/3213/3381/3998 (in Isolates A–D respectively) individual genes evaluated, read counts were highly correlated between biological replicates for all isolates for both the direct and PCR kits (biological replicates *R*^2^ range: 0.90–0.98, Fig. 2). Slightly weaker correlations were observed between technical replicates (*R*^2^ range: 0.76–96, Figs S7–S10). The correlations between technologies were similarly strongly positive (*R*^2^ range: 0.93–0.96) although we observed that these correlations were weaker for genes with a high GC content (here defined as GC content >52%) or low GC content (defined as GC content <44%) (Fig. 3). Strong correlations between read counts were also seen amongst flow cells for biological replicates sequenced using the same kit (Fig. S11). Restricting only to ARGs also revealed very strong correlations between read counts for biological replicates and between kits (*R*^2^ range: 0.93–0.99, *P*<0.001).

### Differences in gene ‘expression’ by kit used

**Fig. 4.**
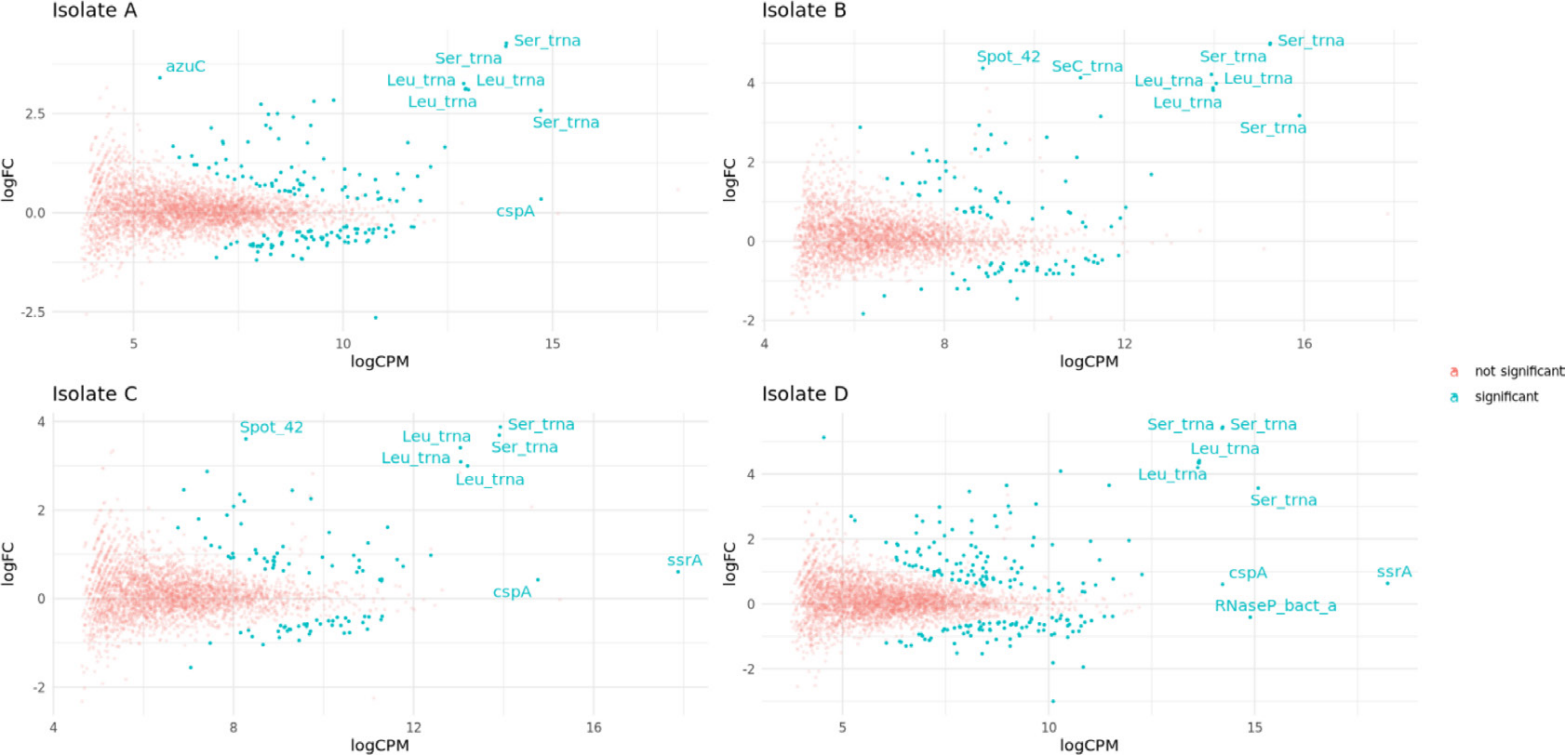
Gene ‘expression’ differences observed between sequencing methods. For each isolate (A/B/C/D), the log count per million (logCPM) for each gene is plotted against the log fold change (logFC) between PCR and direct kits. Genes with significant differences (i.e. significantly differently expressed in the PCR vs direct kit at a *P*<0.05 threshold after a false discovery rate adjustment) are shown in blue. Genes are annotated if they are significantly differentially expressed between the kits and are in the top five ranked genes for either logCPM or logFC. Isolate A: *n*=397 genes significantly differently expressed vs 3389 not significantly differently expressed between methods; Isolate B: *n*=253 genes significantly differently expressed vs 2960 not significantly differently expressed; Isolate C: *n*=217 genes significantly differently expressed vs 3164 not significantly differently expressed; and Isolate D: *n*=488 genes significantly differently expressed vs 3510 not significantly differently expressed.

Across the four isolates, of those annotated genes with reads mapping to them which were not rRNA and after correction for multiple comparisons, 1355/14 378 [9.4%; Isolate A 397/3786 (10.5%), Isolate B 253/3213 (7.9%), Isolate C 217/3381 (6.4%), Isolate D 488/3998 (12.2%)] genes were observed to have significantly different ‘expression’ between the direct and PCR kits (Fig. 4). Of these 14/397 (3.5% Isolate A), 15/253 (5.9% Isolate B), 14/217 (6.5% Isolate C) and 16/488 (3.3% Isolate D) were tRNAs. In comparison only 77/14 378 (0.5%) genes were significantly differentially expressed between biological replicates of the same isolate using the same kit. Overall, only 335/1355 (24.7%) genes significantly differentially expressed between kits had a log fold change (FC) >1 (Fig. S12). In the PCR-based kit, transcripts mapping to ‘over-expressed’ genes were significantly shorter [350 bp (IQR 319–568) vs non-over-expressed 457 bp (385–551), *P*<0.001]. There was no difference in the proportion of plasmid (37/301, 12.3%) and chromosomal genes (1318/14,077, 9.4%) that were significantly differentially expressed between the direct and PCR kits (*P*=0.09). Of the 36 ARGs found in the assemblies, only 12 were expressed in all technical replicates of at least one isolate (Fig. S13). Only two (both in isolate D – blaCTX-M-15 logFC −0.22, *P*=0.002 and sul2 logFC −0.64 *P*<0.001) were significantly differentially expressed between kits.

## Discussion

The ability to sequence long mRNA transcripts using nanopore sequencing offers the potential to identify and quantify transcripts in a single assay, which could help to evaluate the relationship between genotype and phenotype for key clinically relevant traits *in vitro* (e.g. AMR). In this study, using clinical *E. coli* isolates carrying relevant AMR genes, we describe a laboratory and bioinformatic workflow for nanopore RNA-Seq and show that gene expression counts are highly correlated between biological replicates and flow cells, although there is some evidence of significant differences in expression signatures generated for ~5% of genes depending on the kit. Notably, many coding sequences that were differentially ‘expressed’ for experiments using the PCR versus direct kits were tRNAs (Fig. 4), consistent with a known preference for PCR to amplify shorter fragments in a cDNA library mix of variable fragment lengths [1,7]. However, expression signatures in our dataset were similar for both kits, suggesting that PCR-based nanopore RNA-Seq would be a robust way of defining this for specific research questions, bearing in mind this potential methodological bias.

A previous study evaluated direct and PCR-based nanopore RNA-Seq using a single relatively antibiotic-susceptible reference isolate [7], and also demonstrated highly concordant results between methods except for genes with unusually high or low GC content (i.e. <46/>54%). Here, we demonstrate that the PCR kit is also suitable for use with clinically relevant multi-drug-resistant isolates containing multiple plasmid-based ARGs. Whilst we observed statistically significant changes in expression for two ARGs in one isolate, both had small fold-changes (0.64/0.86) and this finding should be treated with caution given the use of only two biological replicates in this study. To our knowledge this study provides the first such data supporting the idea that this methodology is suitable for quantitative transcription analysis in these clinical isolates and the methodology presented could probably be adapted for other related priority pathogens.

The inclusion of only four isolates of the same species, whilst to our knowledge representing the largest evaluation of the use of nanopore RNA-Seq for clinically relevant *E. coli* isolates, is nevertheless a significant limitation. In common with existing literature in this area and primarily due to the very high costs associated with this technology, our study lacks negative controls and so we cannot determine the degree to which the ‘kitome’ may explain some of the variation observed. The decision to use two biological replicates per sample was primarily driven by resource constraints given the relatively high costs associated with Nanopore-ONT sequencing at the moment. Nevertheless, this may reduce our ability to detect smaller changes in expression given that a previous evaluation demonstrated that three or more replicates are required to detect log_2_ changes in expression of ≤1 [17]. Another limitation is that our analysis only captures RNA expression in a single environmental state (growth in antibiotic-free culture medium) which may not represent the eventual use case and is likely to be a simplistic reflection of expression which occurs *in vivo* in patients and may explain why some genes present in hybrid assemblies/reference transcripts were not detected by RNA-Seq. The PCR step in the PCR-based kit, when following the manufacturer’s instructions, has a preference to overamplify short fragments, especially at a higher number of cycles and may introduce bias associated with different GC proportions which should be considered when applying these methods to other species [1,7]. In common with other recent studies [7,13], we found *in vitro* rRNA depletion kits to be imperfect and variably effective; further optimization of this is warranted. Similarly, use of newer R10.4.1 flowcells with updated basecalling algorithms may improve the Q-score distributions obtained in this study. Whilst a recent study [7] demonstrated good correlation between Illumina and Nanopore RNA-Seq results, further work is required to fully understand potential bias introduced by the respective library preparation steps. Finally, the kits and flow cells used in this experiment have been recently superseded by newer flow cells and sequencing chemistries. The direct cDNA sequencing kit is no longer available to buy. Alternatively, direct cDNA sequencing can be achieved using the new Ligation Sequencing kit V14 (SQK-LSK114) with the addition of three oligo primers (VN primer, strand-switching primer and PR2 primer) purchased from a third party. The PCR-cDNA barcoding kit (SQK-PCB111.24) is scheduled to be discontinued from the Nanopore store in May 2024. The recommended replacement is SQK-PCB114.24; although it seems unlikely that this would affect the findings of this study, repeat characterization using our workflow as a framework would be warranted for these and future upgrades, which occur frequently.

In summary, we demonstrate that nanopore RNA-Seq appears to be highly reproducible between biological and technical replicates, with a relatively small number of differentially expressed genes between libraries prepared using the PCR versus direct kits. Potential users need to weigh up the benefits associated with the PCR kit of greatly increased sequencing yield and therefore analytical feasibility with the potential drawbacks, namely a smaller proportion of mappable reads, the apparent generation of shorter reads of a lower quality and a small risk of PCR-associated bias.

## Abbreviations

AMR: antimicrobial resistance
ARG: antimicrobial resistance gene
DGE: differential gene expression
FC: fold change
IQR: interquartile range
MLST: multi-locus sequence typing
ONT: Oxford Nanopore Technologies
PCR: polymerase chain reaction
RNA-Seq: RNA sequencing
ST: sequence type
